# Collectanea

**Published:** 1832-06

**Authors:** 


### COLLECTANEA.

Floriferis ut apes in saltibus omnia libant,
Omnia nos, itidem, depascimur aurea dicta.

### SURGERY.

*Fistula in Ano cured in a Consumptive Patient.* M. Frank, forty-two years of age, a tailor by trade, and of a weak constitution, having a narrow chest, and complaining of severe pains in the thorax when he worked at his business, and having had for the last two years a slight hæmoptysis, reports that, at sixteen, an abscess formed, and opened spontaneously, near the anus, of which the sides became fistulous. About six months after the opening of the abscess, he was admitted into the Hôtel Dieu in Paris, where his fistula was operated on by incision, and in three months the cicatrization was complete.

But in October, 1827, another abscess formed at the side of the anus, on the opposite side to that which had gathered in 1812; like the former, it opened of its own accord, and degenerated into a fistulous sore, which was quickly followed by another. The man consulted a physician, who did not think it advisable to operate, believing that the operation was contra-indicated by the phthisical appearance of the patient, and considering the fistulæ, in fact, as useful means of evacuation, to determine irritation from the lungs to a distant part. Nevertheless, this physician wished to have a consultation on the case; and Amusat, being called in, declared, after an attentive examination, that he could not coincide with his colleague, as he thought an operation necessary; for that, without it, the fistulæ would progress, and destroy the whole of the external opening of the anus, and thus expose the patient, already weakened by his pulmonary affection, to all the dangers of the absorption of pus, of a cancerous discharge, and so forth; without any real benefit to the disease of the lungs, which could not be derived from an issue or a seton.

The operation being determined on, it was performed by M. Amusat on the 2d of December, and, no untoward symptoms occurring, the wounds were healed by the following March. Early in February, Amusat made an issue in the thigh; since which time the man's general health has much improved, his complexion has become clearer, and his eyes brighter; he has an appetite, his strength is increased, and he has not suffered any considerable inconvenience in the respiratory organs, although he is certainly consumptive. Many members of a medical society to which M. Amusat introduced the patient, recognized decidedly cavities in the lungs."—H. A. J. Morere, *Considérations sur les Fistules Stercorales.*

---

*Case of Excision of the Elbow-Joint.* By David Kerr, Esq. Surgeon, Aberdeen. Communicated by Mr. Syme.

Alexander Gray, aged sixteen, was recommended to me in the beginning of May 1830, with disease of the left elbow-joint of ten months' standing. The swelling extended a considerable way both above and below the joint. Suppuration had taken place over the inner condyle of the humerus, and in various places below the joint. The ulcers had degenerated into fistulous openings; and through these a probe could be passed to the lower end of the humerus, which was in a carious state. There was a constant gnawing pain in the joint, shooting down towards the wrist, greatly aggravated by pressure or the slightest motion. His general health was much impaired; his pulse 120, and feeble; he had night perspirations; and, in short, was far advanced in hectic fever. Such were the leading symptoms of the disease when he was placed under my care. To give a detail of its history and treatment from the commencement were superfluous. It may be sufficient to state, that he ascribed the origin of the disease to an injury which he received on the joint when on a voyage to Jamaica, and that it gradually continued to gain ground, notwithstanding a variety of treatment by different practitioners, till at length his health became so much affected, that the hospital surgeon, under whose care he then was, deemed it necessary to recommend amputation, an expedient, however, to which he refused to submit, and accordingly left the hospital.

It was soon after this that he applied to me. To improve his general health he was sent to the country, where he continued to gain strength for several weeks; but after the beginning of June his strength again began to decline, and by the middle of July had sunk so far, that it was evidently necessary to have recourse to an operation, for there was no reason to suppose that he could long bear up under a disease so irritative, and of so untractable a nature. Excision of the joint was therefore resolved on, and, as soon as the nature of the operation and its probable consequences were explained to him, he readily gave his consent. The operation was acccordingly performed on the 23d July, in the following manner:

The position of the patient was that recommended by Moreau, so that the diseased arm hung over the edge of the table, presenting its posterior and outer surface. The brachial artery being compressed by an assistant, an incision was made posterior, but as near to the course of the ulnar nerve as could be ascertained from the diseased state of the parts, commencing about two inches above, and terminating immediately below the joint. A similar incision, parallel to the first, was made on the outer side of the arm; and a transverse section, which divided the tendon of the triceps immediately above its attachment to the olecranon, connected the inferior extremities of the two longitudinal incisions. The flap, consisting of the integuments, diseased cellular tissue, and lower extremity of the triceps, was dissected from the surface of the bone. The extent of disease in the humerus being ascertained, the soft parts adhering to its anterior surfaces were separated from it by the scalpel, which was then passed between the bone and flexor muscles, and retained in that situation till the bone was divided by the saw over the handle of the scalpel, which served as a protection to the soft parts beneath. The removal of the lower portion of the humerus was completed by raising and detaching it from its remaining connexions. The articulating surfaces of the bones of the fore-arm being thus completely exposed, they were found denuded of their cartilage, exhibiting the true carious appearance, and in every respect as much diseased as the humerus. It being therefore necessary to remove the upper extremities of the radius and ulna, the longitudinal incisions were extended about two inches below the joint, and the flap separated, so as to lay them bare. The whole of the ulna above the coronoid process was then removed by the saw, and the head of the radius by the forceps. The diseased parts being thus removed, the wound was sponged out, and as no vessel required the application of a ligature, the flaps were laid down and secured to each other by stitches.

The patient was placed in bed, with his arm supported on a pillow, and simple dressings applied. By the end of three weeks the hectic symptoms completely subsided, and from that time his health improved rapidly. The soft parts being much diseased, the wound healed up slowly. Several of the old sinuses continued to discharge a little for months, and one of them at this date affords a little serous exudation; but from the appearance which it has lately assumed, there is not the least doubt that, in the course of a few weeks, it will be completely cicatrized. The thickening about the joint has entirely subsided, and he retains an arm, which, though necessarily shorter and less perfect than the other, bears no comparison in point of utility to a stump. He can make very considerable exertion with it in lifting weights, can raise it to his head by the power of its own muscles and employ it for many useful purposes, and, what is still more gratifying, it is daily increasing in strength and utility.—*Edinburgh Med. and Surg. Journal.*

---

### TOXICOLOGY.

### *Account of several Cases of Poisoning by Belladonna Berries, occurring simultaneously.* By A. L. Koestler, Vienna.

The author of this memoir having had an opportunity of observing the action of belladonna berries, taken at the same time by persons of different ages, has recorded with care all the phenomena that so rare an occurrence presented, and has reported in detail the history of the cases, of which we shall give an abstract.

A man, accompanied by his son, aged nine years, walking one afternoon in the woods near Dornbach, and seeing the branches of belladonna bearing black and brilliant fruit, resembling wild cherries, he gathered some for his son, who eat them freely on account of their sweetish taste; he also ate ten berries himself, and carried home a large quantity for his other children. Another son, not quite five years old, ate a great number: two elder daughters ate less. All went to bed afterwards, apparently well. Towards morning the two boys were restless, and soon became delirious, and could be kept in bed with great difficulty. A physician was sent for, who made them swallow some soap and water, and ordered them coffee; but they took none.

About ten o'clock in the morning, M. Koestler was consulted, (the author of this paper,) who made the following observations:

A little while after eating the berries, the father drank some new and sourish white wine; during the evening he vomited, and subsequently had several stools. In the morning he suffered only from a slight headach, accompanied with a little stupor, and from time to time he had some griping in the bowels. The youngest of his daughters, who had eaten fewest of the berries, and who had vomited during the night, complained of pain in the head and of dimness of sight; but the pupils were not much dilated. The other daughter, who was older, had eaten more, and she had not vomited as her sister had, but she presented more alarming symptoms: viz. violent headach, with stupor, vision indistinct, pupils very much dilated, walk unsteady and tottering, vertigo, stomach and bowels torpid; pulse regular, tongue clean, and occasionally slight eructations, having the smell of belladonna. In the two boys, the symptoms of poisoning appeared in all their force; restlessness, and attempts to escape, so that they were constantly obliged to be forcibly confined to their beds; continual motions of the hands and fingers, and desire to lay hold of the coverlets, or other objects within their reach, or to thrust their fingers into their nostrils; delirium acute, but the wanderings only on lively subjects; actual vision almost gone, but at the same time both the boys fancied they beheld a crowd of objects; extreme dilatation and insensibility of the pupils; the eyeballs alternately fixed and rolling; spasmodic actions of the muscles of the face, grinding of the teeth, yawning, &c.; voice hoarse and weak; slight swelling of the left side of the throat, and burning sensation in the œsophagus (in the elder of the two boys); decided aversion to all sorts of liquids in both, and spasmodic attacks whenever they were forced to swallow anything; lastly, great excitement of the genitals, (erections, &c.,) and involuntary passage of urine. On the whole, the symptoms presented (as will be seen) some analogy to *mania* without fever; for the vascular system was neither locally nor generally excited, and the respiration was not sensibly disturbed.

Some belladonna berries, and also a small branch of the plant which remained, quickly made the medical attendant acquainted with the nature of the poison.

Two indications presented themselves: the first, to evacuate what poison might still be found in the stomach, (for the frequent eructations shewed that the stomach was not altogether free;) the second, to relieve the symptoms which resulted from having eaten so deleterious a substance. As there were no signs of gastritis present, the first indication was met by the exhibition of a mixture composed of two scruples of ipecacuan, four grains of emetic tartar, and an ounce of water, with a little oxymel of squills and simple syrup, which was ordered to be taken in half-teaspoonful doses every ten minutes, until the desired effect was produced. The author trusted to the vegetable acids to fulfil the second indication. The father and the younger of the two daughters did not take any emetic, as they had only slight headach. The other daughter vomited quickly after the fiist dose, and she, as well as her father and sister, soon recovered, by merely taking lemonade.

The two boys did not vomit until they had taken four scruples of ipecacuan and ten grains of emetic tartar: the eldest then brought up many of the seeds and remains of the belladonna, mixed with mucus and bile; the youngest discharged less. As swallowing was extremely painful, they had clysters of vinegar and water administered every hour, and vinegar lotions were assiduously applied to the head and all along the spinal column. By these means the agitation diminished considerably; nevertheless the youngest slept very little during the night, and both the boys were delirious the following day, although in a less degree. They passed many fetid evacuations from the bowels, in which the remains of belladonna berries were perceived. On the morrow they were well, with the exception of a slight dimness of sight, and a feeling of tightness in the neck of the eldest; the intellect re-established. They had vinegar and water injections morning and evening, and likewise took several warm baths.

These cases afford many data as to the mode in which belladonna acts: they shew that the berries of this vegetable affect the central nervous system exclusively, and that they probably possess a purely narcotic power, while the leaves and the roots contain, as it is said, something more acrid and exciting. By its effects on the organs of deglutition, the fruit of belladonna shews some similarity to the hydrophobic poison, it likewise equally presents certain analogies with the (miasmatic or other) principle of typhous fever by many symptoms.

After the discharge of the deleterious matter, the vegetable acids were employed with success; and in their administration the author followed the advice of Orfila, who recommends their use in cases of narcotic poisonings; and he concludes by asking whether there should not be some therapeutic experiments made with the belladonna berries, as they seem to promise to afford a soothing narcotic medicine, which would not excite any febrile action.—*Medicinische Jahrbücher des k. k. Oesterreichischen Staates.*

---

### *Poisoning with Colchicum.* By Thomas Fereday, Esq.

David Cole, æt. forty-four, a stout muscular man, feeling pains in his bowels, to which he was subject, about six o'clock in the morning of the 8th of March last, swallowed, believing it to be rum, about two ounces of wine of the seeds of colchicum. He immediately discovered his error, but, knowing its effects in a small dose, conceived it would be followed by vomiting and purging sufficient to avert mischief. He sought no medical advice until four in the afternoon, when

I first saw him. He was sitting in a chair, his elbows on his knees, and told me that he felt no inconvenience for an hour and a half after taking the dose, when pains in the bowels came on, but that he continued his labour until eleven o'clock, when pains in his stomach and bowels, retching, and copious vomiting of a yellowish fluid, compelled him to desist.

Four o'clock P.M. He describes the pain in the epigastrium as agonizing, and says it is like a knife piercing him. The retching is incessant and extremely violent, but no fluid is evacuated; there is tenesmus; a small quantity only of fæcal matter has passed. No tenderness upon pressure either in the epigastrium or abdomen. The appearance of the tongue is natural; the pulse small, slow, and feeble; breathing not much affected; the feet cold. His countenance is anxious; features sharp; his cheeks, lips, and palpebræ, purple. Upon attempting to walk, says he thinks he shall loose the use of his limbs.—A mustard emetic was given, followed by copious draughts of warm water and gruel. These were soon returned, with apparently no mixture. Cathartic medicine given, and immediately returned. Was put to bed; warm bricks were applied to the feet, and hot flannels to the stomach. To take forty drops of laudanum immediately; gruel and coffee plenteously.

Nine P.M. The retching, vomiting, and pain in the stomach, continue with undiminished violence; the fluid vomited contains a sediment like coffee-grounds; very thirsty; has made little water. Twenty drops of laudanum every two hours; a blister to the epigastrium; sinapisms to the feet; an enema every hour.

9th, six o'clock A.M. Has passed a sleepless night; the symptoms remain unaltered. The eyes are sunk; the feet warmer; skin generally natural; no perspiration; pulse scarcely to be felt; respiration hurried; great thirst; no urine. Enemata returned without fæcal matter. Camphor, calomel, and opium, every three hours; an effervescing draught, with brandy, every hour.

Eight o'clock P.M. The retching and pains continued until four o'clock, when the bowels were much distended. Has since had copious liquid stools, dark coloured and very offensive, and expresses himself better. Makes a few drops only of urine; loses his sight for a minute or two after getting out of bed to the night-chair. The pulse is scarcely perceptible, and occasionally intermits. He is perfectly sensible, but talks with effort; calls continually for water. Aromatic confection, carbonate of ammonia and camphor mixture, with brandy, every hour.

10th. In the course of the night his stools passed involuntarily, and in great numbers; his weakness increased, and he died a few minutes before five o'clock this morning, perfectly sensible to the last moment.

*Sectio Cadaveris.* The face, neck, upper and front part of the thorax, insides of the arms, front of each fore-arm, and the insides of the thighs, were covered with patches of a purple efflorescence, as were also the integuments of the scrotum and penis. The muscles of the fore-arm were very rigid, and their fibres contracted into hard knobs. The great omentum, instead of covering the front of the intestines, was turned up between the stomach and convex surface of the liver behind, and the diaphragm in front, from the efforts of vomiting. There was increased redness in a portion of the peritoneum covering the jejunum. The stomach and bowels were coated with a thick, tenacious, but colourless, mucous. On a portion of the mucous membrane of the stomach, near the cardiac orifice, and corresponding to its great arch, was a patch of redness about the size of a half-crown piece: its secretion here did not vary in tenacity, quantity, or colour, from that of any other portion of the membrane. Upon dividing it at this part, its section presented nothing beyond its usual appearance; there was no pulpiness, no thickening, but a small quantity of blood was effused between it and the muscular coat, giving the reddened internal appearance. Careful examination of that portion of the reddened peritoneum covering the jejunum, demonstrated the like hemorrhagic condition of the blood-vessels. Blood was effused between the peritoneal and muscular coats, but the mucous membrane corresponding to this portion was perfectly healthy, at least it was perfectly free from inflammation. No other trace of inflammation was observed in any other portion of the abdominal viscera. The gall-bladder was distended with healthy bile. The urinary bladder was contracted and empty.

The pleuræ costales were much reddened. The lungs were of a most beautiful purple colour externally, did not crepitate, and were gorged with black blood, which had become effused underneath the pleura pulmonales in spots of various sizes: these were very numerous about their roots and edges.

The pericardium contained no fluid, nor was it reddened; yet numbers of ecchymosed spots were observed in that portion of it attached to the central tendon of the diaphragm, and also thickly interspersed upon the surface of the heart itself, more especially about the centre of the coronary vessels. Heart flabby, and its structure easily broken down. The cavæ, the right auricle and ventricle, and the pulmonary artery, were filled with black blood, partly coagulated, partly fluid; the left auricle and ventricle empty.

I regret that neither my own entreaties nor the influence of the coroner, were sufficient to obtain permission to examine the head. So little were the functions of the brain disturbed during life, that it is probable no other diseased appearance would have been observed save a participation of its sinuses in the distention of the right side of the heart.

The deleterious effects of a bulb of colchicum are said to depend, in a great measure, upon the season of the year when it is collected; but the seeds, matured only in the spring, must possess uniform properties.

Colchicum is classed by most writers as an acrid or rubefacient poison, and poisons of this class are defined as producing inflammation when applied to the intestinal canal, and, if taken in sufficient quantity, the same effects as the corrosive. No such effects, however, followed its exhibition in this case; for, in addition to the want of the usual post-mortem appearances of inflammation, I consider the patches of redness in the stomach and peritoneum too partial for inflammation from such a cause. The jejunum, too, possesses a singular immunity from disease, as is shewn by the quotation of Andral's table in Mackintosh's Practice of Physic. The hæmorrhagic condition of the system observed here accords with the observations of Dr. A. Thomson, published at page 281 of the Lancet for 1831, who says that colchicum has an effect of producing effusion of blood from all the mucous tissues, the skin alone excepted, and that a peculiar laxity is observable in the cellular membrane, producing a diminution, if not total destruction, of its adhesive powers. The purple efflorescence of the skin, the effusions of blood between the coats of the stomach and of the jejunum, and underneath the pleuræ and the pericardium in this case, all prove the accuracy of these remarks. I feel satisfied that not the slightest inflammation existed in either of the above-mentioned structures, for with respect to the stomach and bowel, where redness was perceptible, the mucous membrance was unchanged in structure; there was neither pulpiness nor thickening, nor did it present a more vascular section than other portions of the membrane: a very important observation, too, its secretion was not changed in quantity or consistence. With respect to the lungs, it was difficult to trace, or rather distinguish, either blood-vessels or bronchiæ: a section of them seemed more like the section of a clot of venous blood, so greatly were they congested with black blood. The state of the breathing during life bore no kind of proportion to this appearance.

Colchicum is also classed as a poison acting upon the nervous system by absorption, and through this medium exerting a peculiar influence on the arterial circulation, and this appears to be its true modus operandi. Sir Everard Home found by direct experiment that its effects were the same, whether taken into the stomach or introduced into the jugular vein, only that in the latter case its effects were more quickly developed than in the former, and hence he concludes that its action upon the different parts of the body is through the medium of the circulation, and not from its immediate effects upon the stomach and intestines. The primæ viæ have been frequently, and perhaps generally, found inflamed where colchicum has been taken in large quantities; but this is no proof of its direct effects upon them, since arsenic will produce inflammation there (the stomach and bowels) when applied to the external surface of the body! Sir Everard Home found that colchicum reduced the pulse in all cases where it was ordinarily used, and this is a material help in enabling us to trace the order in which the three great functions of the body are severally affected by it. It would seem that the heart is deprived of nervous energy, and, in consequence, beats feebly. Now, by the natural process of inspiration, the trachea and bronchiæ, with their minute ramifications, together with the air-cells, are distended with atmospheric air, an elastic fluid. At the standard of health the heart contracts with a force probably three hundred times greater than the force exerted by the pressure of the air in the lungs, and the blood, instead of being impeded, flows more freely; hence the important connexion between the circulating and respiratory functions. We have seen that colchicum, in an ordinary dose, depresses the heart's action. What must then be expected to follow the administration of a dose like that in question, for it does not appear to be one of those remedies, the principle of whose action is altered by its dose! The action of the heart is almost paralized; it can no longer overbalance the pressure exerted by the air in the lungs; the blood can no longer be propelled as usual through their vessels; congestion takes place in the veins; the blood is unarterialized, (and whether its exposure to the action of the fibrillæ of the ganglial system of nerves, in its passage through the arteries, has any influence in effecting this essential change, the lungs are not able to exert their necessary preparatory action); and unarterialized blood gradually destroys the action of every part through which it circulates. The organs of digestion, assimilation, circulation, and secretion, were severally deranged in function, whilst the intellectual and locomotive powers were little affected. This proves the great extent to which the sympathetic or ganglial system of nerves was affected, and the almost immunity of the cerebro-spinal system.

It must be obvious that no treatment in this case, held out any hope of the patient's recovery. When first I saw him, he had swallowed the dose ten hours, when time had been allowed, over and over again, for it to enter the circulation. An emetic, or the stomach-pump, employed before this had taken place, would have dislodged it, and probably have saved his life.

Since poisons act in so many different ways upon the animal economy, some primarily through the medium of the nerves without being absorbed, producing death by suffocation, from paralysis of the respiratory muscles, or by syncope; others by entering the circulation, and exerting their influence on the heart, brain, and alimentary canal; others through the same medium on the spinal marrow; and lastly, others having a purely local action on the mucous membrane of the alimentary canal; and, since this difference in their physiological actions renders a plan or treatment safe and justifiable in some instances, which would be followed by the most tragic effects in others, it behoves us to be aware of the order of the parts of the system through which they exert their influence; for, as each has its own modus operandi, it is by a knowledge of this only that we can arrive at scientific and safe indications of cure. Magendie, for instance, found that a state of depletion facilitated absorption, but that a state of congestion impeded it. How important a knowledge of this fact is in the successful treatment of two familiar poisons, arsenic and corrosive sublimate! Dr. Paris has classed the former with those poisons which produce their effects by absorption, the latter with those having a purely local or corrosive action. Both are followed by inflammation of the primæ viæ; yet the experiments of Magendie teach us, that if we deplete to subdue the inflammatory symptoms when arsenic has been taken, so long as any portion of it remains in the stomach and bowels, we necessarily hasten death by promoting its absorption. On the contrary, corrosive sublimate, having a local action, and not being absorbed, needs no such precaution: we may bleed from the outset.

The same principle is applicable to the poison of colchicum. It produces its effects by absorption. We should therefore be very cautious how we bleed to subdue any inflammatory action of the stomach and bowels, until the poison has been completely ejected from them.—*Med. Gazette.*

---

### BOTANY.

*Influence of Musk on Vegetation.* Dr. Goppert, of Breslau, having ascertained the curious fact, that all odorous vegetable principles, as the essential oils, camphor, &c. have an extremely noxious effect on vegetation, arresting totally the development of plants with which they come in contact, and finally killing them, was desirous of ascertaining whether the odorous animal substances would have a similar effect; for this purpose he used musk. The vegetables were exposed to its action in two ways; either the roots of the plant were moistened with water, holding musk in solution, or else the whole plant was exposed to the exhalations of a considerable quantity of musk for a long time, generally until the plant grew so large as to render it necessary to stop the experiment, the vessel used being too small to contain the plant.

In no case did this animal substance appear to exert any injurious influence on vegetable life. A great number of plants of different classes were experimented on, but all with similar results. He found, however, that the plant, although taking up the water in abundance, did not absorb any of the musk which existed in solution, nor did the tissue of any of the vegetables exposed to its action exhale its characteristic odour.

He also determined that those parts of plants, as the cicatricula of the Berberis, the leaf of the Mimosa, &c., which possess motion, and a kind of sensation, did not lose these vital properties by the action of musk.

He reasons from this inertness of musk, that the opinions concerning the nature of its odorous principle cannot be correct: Guibourt considering it to depend on a volatile oil, and Buchner on carbonate of ammonia; the exhalations from both which substances are highly noxious to the life of plants.—*Zeitschrift fur Physiologie*, 3 band. 2 heft., and *Dublin Journal of Medical and Chemical Science.*

---

### MISCELLANEOUS.

*Electro-Magnetism.* We mentioned in our last the brilliant results of Mr. Faraday's experiments in proving the similitude if not the identity of electricity and magnetism, and that he had been enabled to procure an electric spark from magnets. These magnets, however, were artificial magnets, i. e. bars of iron rendered magnetic by the induction of galvanism; but, since our last number, we have seen him exhibit a spark produced from a natural magnet, and have indeed ourselves repeated his experiment with the very powerful natural magnet which Professor Daniell has secured for the laboratory in King's College. The means by which this brilliant result is achieved are simple in the extreme: the two poles of the natural magnet are connected by a moveable bar of soft iron, from each end of which proceeds a wire, the extremities of which come almost in contact, and are so arranged that they strike on each other whenever the communication is made or broken between the two poles of the natural magnet by the bar of iron; and at every contact a brilliant and beautiful spark is seen.

Professor Ritchie has even succeeded in inflaming a mixture of oxygen and hydrogen gasses by means of this spark.

